# The Influence of Tropical Climates on European Constitutions

**Published:** 1842-04

**Authors:** 


### ART. IV.

1. *The Influence of Tropical Climates on European Constitutions.* By JAMES JOHNSON, M.D., Physician to the late King, &c., and JAMES RANALD MARTIN, Esq., late Presidency Surgeon, and Surgeon to the Native Hospital, Calcutta. Sixth Edition, revised and greatly improved. —*London,* 1841. 8vo, pp. 693.
2. *Researches into the Causes, Nature, and Treatment of the more prevalent Diseases of India and of warm climates generally.* By JAMES ANNESLEY, F.R.S. F.S.A., late President of the Medical Board of Madras. Second Edition.—*London,* 1841. 8vo, pp. 606.
3. *The Madras Quarterly Medical Journal.* Edited by SAMUEL ROGERS, Assistant-Surgeon, Madras Establishment. Vol I. for 1839. Vol. II. for 1840. 8vo, pp. 486, 462.
4. *Transactions of the Medical and Physical Society of Bombay for the year* 1840.—*Bombay,* 1840. 8vo, pp. 224.
5. *The Indian Journal of Medical and Physical Science.* Edited by FRED. CORBYN, Esq., Garrison Surgeon of Fort William.—*Calcutta,* 1840.

THE subject of tropical disease is one which the prodigious amount of our intertropical colonization renders deeply interesting to us; and it is satisfactory to us to add, that our medical literature regarding it is, as it ought to be, extensive and valuable. The latter fact is evinced by the works now before us; two of them showing by the editions they have passed through that they are stamped with the seal of public approbation; whilst the Medical Transactions and Journals show the probable progress of this branch of literature in the hands of the British medical residents abroad, who display so much activity in collecting information and communicating it to their contemporaries and successors. We consider, then, that we have before us the labours of two generations; those, "rude donati," who have earned reputation by intertropical research, and the younger aspirants for fame, who manifest a disposition to follow in those paths which their predecessors have so honorably trod. A complete analysis of works so voluminous our limits preclude, but we cannot allow them to pass through our hands without some consideration of the more important portions of their contents.

As might be supposed, the etiology, symptoms, treatment, and mortality of acute disease form the most prominent subjects of these volumes. On all these points we find much valuable information, and much that we feel disposed to transfer to our pages; but that on which we feel at present most disposed to dwell, not more from its importance, than from the obscurity which still overshadows it, is that of the causes of tropical diseases. The writers before us, we are happy to observe, have entered on this branch of their subject with a full perception of its difficulties. One of them, Mr. Martin, the collaborator of Dr. Johnson in this the sixth edition of his work, places these difficulties prominently before the reader in his preface.

"An enquiry into the external causes of disease in hot climates, and a reference to the topographic and statistical facts detailed in the recently published Reports on the health of the British Army and Navy, must satisfy any person that some of the modes of investigation hitherto pursued are insufficient to a full or accurate illustration of those all-important questions; for, so many exceptions have been found in the East and West Indies, but especially in South America, to the reputed causes of many diseases, as to make it no easy matter whether to determine in favour of the rules commonly received, or of the numerous and extended exceptions to them." (Preface, p. vi.)

Heat as a cause of disease. How far heat is abstractedly a cause of disease is an important consideration, and one which does not appear to us to receive that formal attention from Mr. Annesley, at least, which it had a right to claim. Major Tulloch, in his West Indian Report, it will be remembered, expresses the opinion founded on statistical evidence, that mere heat has little influence in the production of disease, though he is disposed to attribute power in this way to heat cooperating with moisture. The narrative, however, by Messrs. Mouat and Henderson, of the march of the 13th regiment of foot from Nuddeah to Berhampore, in 1826, which will be found in the second volume of the Madras Quarterly Journal, and which is copied in the work of Messrs. Johnson and Martin, shows the abstract power of this agent in the eastern hemisphere to be very great indeed. The left wing of the regiment marched from Nuddeah for Berhampore, a distance of sixty miles. Dr. Mouat, who accompanied it, suggested that the marches should take place at night, the weather being very sultry. Though this suggestion was adopted, there was great distress during the march, and sixty-three men became dangerously ill, but no deaths occurred. The right wing, accompanied by Dr. Henderson, moved on the 3d of May from Nuddeah. The same suggestion relative to marching at night was given as in the instance of the left wing. It was not, however, so successfully acted upon, for the camp ground was not reached till after the rising of the sun, which immediately after daylight shone forth with overpowering splendour, whilst an excessively hot wind added to the effect. The day, we are informed, closed with the loss of eighteen lives, and left sixty-three sick in hospital. A halt of one day was indispensable.

The party reached Berhampore on the 8th. Three more fatal cases occured during this period, constituting a total of 21 deaths. The greater part of the sickness and all the mortality took place in a detachment of 278 recruits, who had recently joined the regiment. The old soldiers suffered little: natives not at all. The symptoms are thus stated by Dr. Henderson:

"They complained of difficult breathing, with a sense of tightness and oppression about the chest, followed by giddiness, burning heat of the eyes, and a sense of general fulness about the head, in many amounting to excruciating pain, succeeded by loss of sense and motion, faltering of the tongue on attempting to speak, fulness of the eyes, dilated and fixed pupils, violent twitching of the muscles of the face, particularly those about the mouth, subsultus tendinum, and involuntary stools. Along with these symptoms, the patients had a strong, full, and frequent pulse, tremendous throbbing of the carotid and temporal arteries, flushed, swollen, and sometimes livid countenance, and throughout, a parched and burning skin." (Madras Journal, vol. ii. p. 326.)

In relation to treatment it was observed that two individuals who were largely bled, became convulsed and died, and after death it was found that, though the heart was empty, the vessels of the head were loaded with blood. It was thus "clearly indicated that whatever it was that excited the heart's inordinate action, bloodletting would not subdue it; for as long as a drop of blood remained, it was sent to the head." The plan of treatment ultimately and successfully adopted was the employment of moderate bloodletting, cold affusion, cold water permanently applied to the head, and mercurial purgatives. One very obvious portion of this treatments, cold water to the head, we have ourselves seen employed by the peasantry of Spain and Portugal under similar circumstances; and we have likewise observed in analogous cases, even in this country, more relief from cold to the head, and prompt local bleeding from it, than from any quantity of blood drawn from the general system.

Dr. Murray, inspector-general of hospitals, has some valuable remarks respecting the influence of heat in the same journal:

"It appears to me," he remarks "that few (if any) authors have made us fully aware of the extent to which simple exposure to high temperature causes febrile disease in India; or have given the history of the symptoms proceeding therefrom with requisite precision; most of them having mixed up their description of such affections with the inflammatory varieties of paroxysmal fevers produced by terrestrial (vegeto-animal) exhalations concomitantly with other causes. I have heard doubts expressed as to the nature and proper name to be given to morbid affections arising from high temperature; and I apprehend there is an essential pathological difference between those arising from exposure of the bare head to the direct rays of the sun, or from exertion under strong heat, and such as are induced by the influence of high atmospheric temperature *in the shade.* In the former case, strong excitement is at once produced in the organ affected by the direct stimulus of the cause. Thus, in the case of exposure of the bare head to the sun, the scalp being protected by the hair, and peculiarly constructed, does not blister as the skin of the other parts of the body would do, if exposed to strong solar influence; but its temperature becomes exalted, and the caloric traversing through it and the skull, gives immediate rise to active hyperæmia and inflammation of the contents of the cranium. In the latter case, the application of the morbific agent being on the general system, the cerebral affection which results appears in a different manner: at first there is nervous depression or collapse, and sanguineous congestion, on which reaction follows." (Madras Journal, vol ii. p. 337.)

The affection arising from insolation, Dr. Murray regards as encephalitis, and, in many instances, we believe, correctly; but in others, which we have ourselves witnessed, though not in India, the promptitude of recovery has convinced us that the vascular condition did not pass beyond the limits of hyperæmia. He is of opinion, too, that there seems no doubt that fevers of remittent character, analogous to yellow fever, are frequently induced from the simple influence of intense solar or atmospheric heat; and that these diseases bear a resemblance to those arising from marshy lands, but they are not identical with them. This is an important remark, for it tends to illustrate the occurrence of diseases, such as are ordinarily ascribed to the influence of malaria in localities where the commonly reputed sources of this poison are not discernible. It is well known, and we have already remarked upon it,* that the appearance of what was supposed to be marsh fever in situations where no vestige of marsh was discernible has tended considerably to unsettle our opinions regarding malaria. This remark of a writer of so much experience and acuteness as Dr. Murray, renders it at least probable that a further investigation of the real character of the diseases prevailing in such localities may throw light on the obscurity at present existing on the subject.

Among other morbific causes, malaria receives an ample share of attention in these volumes. Mr. Annesley has a long and valuable chapter on the subject, comprehending a description of the situation, soil, and vegetation, calculated to engender the poison. We do not recommend it to especial notice for any novelty in the facts or opinions,—indeed the writer seems sedulously to avoid doubtful or debatable ground; but for its comprehensive, and we would say, to the student, very instructive view of the ordinarily received doctrines. On one controverted point only does he touch, that of salt marshes.

He remarks that the copious extrication of unwholesome effluvia from salt marshes and partial inundations of the sea has long been admitted, and has only been disputed by one writer of eminence, who instances, in disproof of the position, the salt marshes of one particular district in the western hemisphere. He considers, however, that there is probably some peculiarity, in this case, in the soil and its productions, such as its consisting of a deep bed of sand or gravel but imperfectly covered with vegetation, constituting it an exception to a general rule. But where the soil is deep, of a rich, dark, or clayey mould, or in any respect absorbent, and still more so if it be covered by a rich, rank, or succulent vegetation, and not admitting of a speedy drainage of the waters which may inundate it, insalubrious exhalations are copiously formed; and on all occasions of inundations by the sea, the formation of deleterious effluvia in such situations is abundantly increased. Examples of devastating pestilence from such a cause are frequent at the mouths of the Ganges, the Irrawaddy, and the Indus, and numerous other places in India. This very deleterious effect of salt water, Dr. Annesley does not ascribe to any special septic tendency of salt in small proportions; nor does he speculate on any peculiar chemical decomposition it may undergo or cause in other substances; but he thinks it owing to the quantity of animal matter sea water contains, which occasions it to run faster into putrefaction than fresh water, when subjected to a warm temperature and *kept at rest.* This latter point, *the keeping at rest*, is evidently essential to the pernicious effect. On this point we have the following remark from Dr. Johnson:

"But in May and June, when the rivers are shrunk far within their autumnal boundaries; when the heat is excessive; and when the tides are so rapid, that the bore, as it is called, rushes up past Calcutta, sometimes with the amazing

* See Major Tulloch's West Indian Report, p. 102; and British and Foreign Medical Review, vol. VIII. p. 216.

velocity of twenty miles an hour, not entirely stopping till it reaches Nia-serai, thirty-five miles above the capital; then, indeed, at low water, each side of the river presents a broad shelving slope of mud and mire, covered with vegeto-animal remains, in all stages of putrefaction, and disengaging the most abominable stench; yet no ills whatever are produced by such exhalations." (Johnson and Martin, p. 92.)

The writers before us prudently abstain from speculations on the *nature* of this great morbific agent of tropical regions. Dr. Annesley's expressions on this subject depict very correctly our knowledge, or rather ignorance regarding it:

"The sum of our knowledge," he remarks, "of the nature of this poison seems to be, that it proceeds from those elements which exist in a rich soil and nourish the vegetable and animal kingdoms; and that these elements, when subjected to the action of the sun, together with the influence of the air and moisture, form new combinations, which are volatilized by the sun's heat, and readily combine with the moisture present in the lower strata of the atmosphere."

We approve of this cautious and general expression; but yet we would suggest that Professor Daniell has pointed out a path of investigation which, we trust, our intertropical brethren will feel it their duty to pursue.* What this gentleman has published regarding the sulphuretted hydrogen in the waters of western Africa; its probable dependence on the decomposition of sulphates, existing either in sea water or the soil, by the carbonaceous matter of decayed vegetables; and the apparent connexion between the generation of such gas and the occurrence of fever and mortality, as evinced during the expedition into the interior of the Niger, by Messrs. Macgregor Laird and R. B. Oldfield, though it does not suffice to identify sulphuretted hydrogen and malaria,—shows that this gas is at least a frequent concomitant of the poison, and further investigation may prove it to be something more. Might not the soil of places like Up-Park Camp in Jamaica, and Maroon Town in the same island—the relative sickness of which presents striking anomalies, according to the ordinary doctrines of malaria—be subjected to chemical investigation, with the prospect of explaining the anomalies, and of confirming or refuting the opinions of Professor Daniell? In the former situation, with an *apparent* exemption from the sources of miasmata, 140 per 1000 die annually; in the latter, surrounded, according to received opinions, with the sources of disease, the mortality is but 22 for 1000 in the same period.† A chemical analysis of the soils of places so strongly contrasted might have an important bearing, either in the way of refutation or confirmation, on the Professor's theory. Were this a mere matter of speculation, we should be less earnest on the subject, but the familiar chemical fact that sulphuretted hydrogen and chlorine gas cannot coexist, gives it an important and practical character.

Both of the systematic works before us treat of the predisposing causes of intertropical disease, those causes which are auxiliary to the great

* See Professor Daniell on the Spontaneous Evolution of Sulphuretted Hydrogen in the Waters of the western Coast of Africa and other localities, in the London, Edinburgh, and Dublin Philosophical Magazine, for July, 1841, and Nos. 43 and 44 of the Medical Gazette.

† Tulloch's West Indian Report, pp. 102-3.

vegeto-animal source, in the same tone. Both speak of the depressing passions as very materially instrumental in inducing disease, and their influence is illustrated in Dr. Johnson's work, by striking and instructive examples. By both is indolence and vacuity of mind considered as auxiliary to disease, and its opposite condition as a preventive. Dr. Annesley expresses his sentiments on this head happily and judiciously :

"A calm, confident, and well-employed mind, moderately occupied and interested in the objects of its pursuit, unruffled by vicissitudes of temper, and undisturbed by inordinate indulgence of its desires, with a moderate but sufficient gratification of its wants or wishes to give a foretaste of more perfect fruition, and to leave still more to hope for and to aspire after, so that its capacity of gratification be not exhausted, or its means of enjoyment diminished, is, upon the whole, that state of mind which most successfully opposes the causes of disease."

This is perfectly true ; but Dr. Annesley must be aware that he has depicted both a mental constitution and an assemblage of circumstances which his own knowledge of mankind and the world must have taught him are rather the exception than the rule, and to enjoy such safeguards from disease has hitherto been the lot of few, whether at home or between the tropics. We find no fault, however, with his indicating what all should aim at, though but few attain. All the writers concur both by example and precept on the necessity of temperance. Dr. Annesley expresses himself on this head with his usual clearness and succinctness. He says, that the diet should be nourishing but not heating; animal food should be taken sparingly, and spirituous liquors and strong wines entirely avoided. The lighter and thinner wines may be resorted to in moderation, but excess even in them should be shunned.

From the consideration of morbific causes we pass to that of the diseases induced, in the first place passing in review the sentiments of our authors regarding a malady which, we have occasion to know, ought not to lose its interest in the eyes of the European observer.

Cholera. In the present volumes we do not find much on the subject that has not been said before; but we find some of the previously existing information arranged in a more statistical and convincing form. It has ever appeared to us a matter of importance to decide, whether the epidemic which pervaded Europe in 1832 and a portion of 1833 was identical in character with the disease of India, from which it was generally supposed to be an offset. We were impressed with the conviction at the time of the prevalence of the epidemic in England, from a perusal of the Indian Reports, that there was a difference between what we there read and what we were then observing, and this on a very important point of the English disease, the diarrhœa. We can declare, from an experience founded on more than a thousand cases, that serous diarrhœa, in which the discharges, after the evacuation of the fecal contents of the intestines, possessed the rice-water character observed in the more advanced stages of the disease, was the first symptom which occurred. To this rule we found no exception in cases of which we had an opportunity of tracing the progress. This diarrhœa might precede the other symptoms by but a few hours ; but sometimes it did so by two or three days, the variation depending apparently on the degree of diarrhœa and the strength of the patient. Dr. James Johnson apparently takes the same view of the initiatory symptom, for he says, from an extensive observation of cholera, as it appeared in England, he is perfectly satisfied that the disease is "a serous hemorrhage from the bowels,"—a phrase, the grammatical accuracy of which may be cavilled at, but which depicts very exactly its early stage, and explains extremely well its subsequent character. The experience of the writer of the article Cholera, in the Cyclopædia of Practical Medicine,* which he wrote, as he informs us, from observation of the disease when prevailing epidemically in the north of England, has led him to the opinion, that serous diarrhœa is the first stage of this formidable disease, and that due attention to this stage will prevent much subsequent mischief. Observation of the disease, in India, however, gives different results in relation to diarrhœa. These have been extracted from the Indian Reports, and presented in a tabular form in his book by Dr. James Johnson. From this valuable table we learn, that of sixty-four writers whose opinions it embodies, five only mention diarrhœa as having preceded the other symptoms; whilst a considerable majority of the other fifty-nine explicitly declare that there was no precursory diarrhœa; and, it being only justice to those who omit all mention of it to suppose, that had it occurred it would have been recorded, it follows that there was a very material difference in the disease in the two localities, India and England. How far the difference may be considered specific, or sufficient only to constitute the Indian and European diseases varieties of one and the same malady, we should hesitate to decide.

If this view, that cholera in this country consists in its first manifest symptoms of a discharge of serum from the intestines, be correct, the question arises, on what condition of the intestinal exhalents or of the general system is this discharge dependent? We profess ourselves unable to answer this question, and we know of no positive researches into the disease which can aid us in doing so. Suggestions, however, have been thrown out by Dr. Prout, which, proceeding from an individual so singularly learned and ingenious, we think not undeserving of notice. This gentleman remarks,† that of all the causes which produce forms of disease connected with the development by the secondary assimilating process of oxalic acid and the other indefinable [acid ?] principles down to lactic acid, malaria is the most frequent. He adds, moreover, that at the time of the appearance of the cholera in London, he observed a cessation of the common lateritious sediments from the urine under circumstances calculated to produce them, whilst this fluid presented the character distinctive of the oxalic acid diathesis. At the same time there was remarked an unusual acidity of the saliva and the cutaneous exhalations. Coinciding with these phenomena, Dr. Prout observed a positive increase of the weight of atmospheric air, similar to what might be supposed to be produced by the diffusion of a *heavy gaseous principle* through the lower region of the atmosphere. So far proceed the remarks of Dr. Prout: we can confirm them as to the acidity of the cutaneous exhalations in those actually labouring under cholera, and likewise in another malarious disease, dysentery.

On the subject of contagion, as relates to the disease in India, there

* Dr. Brown, of Sunderland; Cyclopædia of Practical Medicine, vol. i. p. 388 et seq.
† Prout on Stomach and Urinary Diseases, 3d edition, pp. 20-1.

seems little diversity of opinion there. Mr. Annesley thinks that "all the occurrences which, at first sight, seem to favour the doctrine of contagion," may be explained on another principle. Dr. James Johnson gives a valuable summary of opinions on the question in the table we have already adverted to. One witness only thinks it decidedly contagious; one suspects it to be so; one is doubtful; one leans to contingent contagion; one regards it as endemic and contagious; another as being slightly contagious; one is inclined to think it contagious; another as sometimes communicable; whilst two regard it as infectious. Thus ten are imbued with various shades of contagious doctrine; whilst thirteen declare that it is decidedly not contagious. To this discordant list are to be added forty-two who make no allusion to contagion, and, as Dr. Johnson remarks, since the queries of the Madras Board particularly directed the attention of medical officers to the subject of contagion, we may fairly conclude that, where no allusion to contagion is made, no proofs of it presented themselves to the observers. This reasoning appears conclusive, and it follows that the testimony against contagion is, at least, as five to one, (55 to 10.) Of the forty-two parties who make no allusion to contagion, the great majority speak of it arising from some atmospheric or terrestrial cause.

We do not find much to extract on the subject of treatment. As relates to the disease in England, Dr. James Johnson expresses an opinion perfectly coinciding with our own—that by due attention to the first stage of the disease, that of diarrhœa, all the subsequent mischief will be prevented. The same opinion was long ago expressed in the article in the Cyclopædia of Practical Medicine, already referred to. The truth appears to be, that influenced probably by the Indian Reports, which, treating of something different from what we had to deal with, dwelt only on the treatment of the disease in the stage of spasm or collapse, our proceedings in cholera were at total variance with the principle observed in all other diseases; instead of dealing with the initiatory stage where we had power, our attention was concentrated on the appalling symptoms of the advanced or even closing scene, where we were powerless or nearly so.

With regard to the treatment of the disease in India, we meet little meriting notice, excepting a sensible and very decisive remark by Dr. French, in his Report on Cholera in the 49th regiment, in the second volume of the Madras Journal. This gentleman says that, "good nursing is of the greatest moment, from the onset to the termination; and that when patients do recover from a severe attack, more is to be attributed to the kind assiduity of the attendants, and to the judiciously sparing use of medicine, than to the trial of a farrago *of everything that can be thought of.*" Dr. Murray thinks very favorably of the effect in the advanced stage of hot saline enemata, deeming them the only means possessing any influence over the state of collapse. The ingredients employed are: common salt one ounce, carbonate of soda or potash one drachm, and of water at 120° Fahrenheit one pint. An enema of this kind is given every half hour, hour, or two hours, till the circulating powers are excited into reaction, and the pulse rises to a satisfactory degree of strength. Dr. Murray remarks, that he does not consider this remedy as infallible; a proposition which we see no reason to controvert, but which, on the other hand, is strongly confirmed by the Report of Mr. Morgan, surgeon of her Majesty's 57th regiment. This gentleman gave the saline enemata an extensive trial, and then came to the sober conclusion, that "saline enemata are probably as good as any other single remedy; but they are no specific, and they are not to be relied upon for a cure of an intense case of the disease."*

We have enlarged the more in our extracts relative to cholera from a conviction that the disease ought not to be considered unimportant, even in this country. It is true that in contradiction to the prophecies of the class, whom Dr. Johnson sneers at as choleraphobists, we have had no epidemic of the disease since 1833; but we can truly state, that not an autumn has passed since that period without our seeing cases identical in character with the epidemic, and of these cases there has been a proportion fatal. They have never been sufficiently numerous to be considered as more than sporadic; but it has appeared to us that the serous disease has, since the epidemic, very much taken the place of our ordinary autumnal bilious cholera.

SWEATING SICKNESS. Closely allied, as it appears to us, to the worst form of cholera, and bearing a close relation to the *febres intermittentes malignæ*, and *algidæ* of Torti and our own Morton, is the Malwah sweating sickness, described by Dr. John Murray, assistant surgeon, Bengal Horse Artillery, in the second volume of the Madras Journal. England is now happily, from its improved civilization, exempt (with the recent exception of the epidemic of cholera) from those intense pestilences which besieged our ancestors, and there is a degree of interest in being carried back to the forms of disease with which English physicians from the days of Caius, and probably earlier, to those of Sydenham and Morton, had to combat. Dr. Murray, who is evidently a man of learning and talent, has selected for this disease, and we think appropriately, the name "Sweating Sickness," so famed in English story. He shall describe it himself:

"The symptoms of this disease commenced with rigors or chilliness, followed by dull headach, increased heat of skin, and dilated pupils; there was at the same time a burning sensation at the epigastrium, with restlessness and thirst, and, generally, copious watery motions smelling like the flesh of carnivorous animals slightly tainted. In many cases there was vomiting of a similar fluid, attended with cramps in the extremities; and the skin soon became bathed with perspiration. There was also great oppression in the breathing, with anxiety and uneasiness at the precordia, and a weak rapid pulse. At the commencement there was prostration of strength, with a feeling of exhaustion; and afterwards there was real debility, sometimes extending long into convalescence. In the severest forms of the disease all bodily uneasiness soon ceased, excepting that arising from the thirst and pectoral oppression: the perspiration continued excessive and became cold: the mental faculties remained clear till towards the end, when coma gradually supervening, death sometimes ensued within ten hours of the period of attack. *Vomiting and cramps were neither constant nor prominent symptoms;* but in the severe cases, no urine was passed, nor was there any bile in the evacuations till reaction ensued.

"When the disease took a favorable turn, the pulse became more full and slow; the burning heat at the epigastrium, and the precordial oppression diminished; some dark-green feculent matter was passed by stool; a little urine was secreted; and the patient slept. If the case did not proceed at once to

* Madras Quarterly Journal, vol. i. p. 224, &c.

convalescence, the pulse did not become natural, the pupils remained sluggish, there was anxiety, and the skin continued muddy and strongly perspiring.

"After a remission of twenty-four or forty-eight hours, sometimes anticipating by two hours, the same train of symptoms was apt to be renewed. The skin became dry at first and sometimes hot; the burning sensation in the epigastrium recurred, followed by two or three watery nauseous stools, and great exhaustion of the strength; and although the skin became cold, the perspiration increased. There was occasionally wandering of the mind, but extreme collapse, with a state approaching to coma was more common; and these increased after each periodic exacerbation or paroxysm, if the case was proceeding unfavorably." (Madras Quarterly Journal, vol. ii. pp. 80-1.)

There were never any cramps after the first attack, and vomiting was also less frequent. As the disease went on, remissions succeeded the exacerbations or paroxysms with a regular periodicity. When the patient was to recover, the attacks became more slight, and sometimes convalescence was rapid without leaving any organic derangement; but when the disease was of a dangerous character, the collapsed and comatous states were more prolonged after each exacerbation, and sometimes the patient never rallied after they came on.

The blood was found very liquid when venesection was employed, and the serum muddy and mixed with the colouring matter of the red globules, and leech-bites were apt to ooze for a long time in consequence of this dissolved state of the blood. The editor of the journal contrasts this state of the blood with that observed in cholera, remarking that in the latter disease it is thick and dark, resembling treacle or tar, and quoting M. Lecanu to show that the solid matter of cholera blood is sometimes double that of the healthy proportion. In the blood, he says, of patients labouring under typhus, malignant remittent fevers, and scurvy, there is a deficiency of the solid ingredients. The appearances on dissection were a dissolved, dark state of the blood, serous effusion on the brain, and abnormal sanguineous accumulations in the thoracic and abdominal viscera; but, the author adds, the most remarkable morbid appearance was the great alteration in the blood from its healthy state. He is disposed to regard this condition of the blood as the first effects of the application of the pestilential agents, and as the cause of the suppression of the secretions. The editor demurs to this conclusion, and considers that the first action of the poison is on the apparatus of innervation. For our own parts, we think the author's theory *at least* as good as the editor's, and that, therefore, according to a well-known rule, it ought to have been left undisturbed.

The treatment of this formidable disease is discussed by the author with much diffidence, and with a degree of judgment which, we would not say renders the former quality unbecoming, but which shows, at least, that a diffident tone is no index of demerit. He speaks with much doubt of general bleeding, and says, that when had recourse to in the stage of collapse to relieve congestion, diffusible stimulants and tonics, with mild nutrients, require to be employed at the same time, to rouse the vital functions, excite the heart's action, support the strength, and regenerate the blood. With regard to these mixed proceedings of (what we consider) antagonist measures, of which we saw something from the hands of Indian practitioners in cholera, and of which we have heard much more, we do not hesitate to declare, that our observation, so far as it extends, confirms what our reason has told us, that the respective portions of the treatment being appropriate to opposite conditions of the system, when they are simultaneously employed, one at least of them is improper, and often does more mischief than the other can counterbalance. In the early stage of our cholera experience, when we regarded ourselves as little more than the pupils of Indian practitioners, we saw patients sink to rise no more under bleeding, from which, we had been told, marvels would be accomplished in collapse, provided a flow of bright blood could be obtained. We never, however, saw the bright blood in such cases, but we saw an increase of collapse against which stimulants were powerless. During the stage of reaction, leeches were, during the prevalent sickness, very properly applied to the nape of the neck or temples, when the head was much affected; over the region of the heart when there was much oppression there; to the right or left hypochondrium when the spleen or liver was enlarged; but, the writer adds, in several instances, the symptoms on account of which they were applied, disappeared so rapidly after a trifling abstraction of blood, that the utility and necessity of this particular agent was often doubtful, more especially as favorable results frequently ensued where it was not employed. That applied externally by bottles of hot water, and internally in the form of the hot saline enemata, already mentioned, was a powerful agent in supporting the heart's action. Wine given in small quantities with arrow-root, or effervescing draughts, appeared to support the strength under exhausting perspiration. Counter-irritants were useful when the pain in the epigastrium was great and when coma supervened; and of these, nitrate of silver, blisters, and sinapisms were the best. After remission had been established, quinine was (as might be supposed) the most valuable remedy for preventing the recurrence of the primary symptoms and moderating their effects.

FEVER. Mr. Martin remarks,* "that it cannot be too much impressed on the Indian surgeon, that it is on his careful attention to the phenomena of fever, that nine tenths of his usefulness depend;" and certainly all the writers before us show by the accuracy of their descriptions that their own labours have been influenced by this important principle. We should consider ourselves as deviating from the purpose of this article, which is rather to touch salient points than to furnish an analysis of the voluminous works we are dealing with, were we to do much more than impress on our readers, and particularly on students, who may have a prospect of being engaged in the eastern hemisphere, the great extent, and, we would add too, the accuracy of the information regarding the general features of the endemic fever of India, to be found in the pages of Dr. Johnson and Messrs. Martin and Annesley, and the insight which the regimental Reports, published in the Transactions and Journals, afford into the varieties of this staple disease; and, consequently refer them to these works, as preparatory to that experience, the fruits of which, however hardly earned and slowly gathered, constitute the only substantial and available knowledge a medical man is ever doomed to possess.

One document, however, in reference to the treatment of remittent fever which Mr. Martin has taken the trouble to compile, we consider so

* Johnson and Martin, page 125.

valuable, that nothing but want of room prevents us from quoting it, and commenting upon it at large. He has condensed into one view the practice recommended in this disease, whether existing in the East Indies or other warm regions, by various authorities for two centuries. His authorities are arranged in chronological order, and stand thus : 1629, Bontius; 1751, Cleghorn; 1757, Bogue; 1757, Huxham; 1760, Huck; 1768, Pringle; 1790, Balfour; 1795, Chisholm; 1797, Clark; 1799, Blane; 1808, Dr. Robert Jackson; 1810, W. Ferguson, inspector-general; 1811, Bancroft; 1813, Dr. James Johnson; 1816, Sir W. Burnett; 1818, Ballingal; 1819, Dickenson; 1827, Geddes; 1828, Annesley; 1832, Mr. Twining; 1833, Dr. Joseph Brown, in Cyclopædia of Practical Medicine, and, 1835, Dr. Copland, in Dictionary of Practical Medicine. The remedies recommended by all these authorities are stated by Mr. Martin, and, with the exception of those writing towards the close of the last century, it is striking how great is the accordance among them. Excepting Balfour, Chisholm, and Clark, who wrote during the last ten years of the 18th century, all recommended bleeding. The cause of the exceptions is manifest enough; the medical world was then under the sway of the absurd Brunonian reveries, and debility, direct or indirect, daunted the imaginations of all practitioners. Good sense, however, speedily resumed the rule, and between the practice of the earlier writers, from Bontius to Pringle, and those within the 19th century, the difference is in detail, not in principle. There is some difference in the view with which mercury is given, some purposing to affect the system by it; others merely looking to its purgative effects; some give bark during the remissions, others omit it; but the general antiphlogistic and evacuating plan is that recommended by all, with the exceptions stated.

DISEASES OF THE LIVER. These occupy of course a large space in these volumes. The reasons which we have assigned for our cursory view of the subject of fever must equally prevent our traversing the whole of the present ground with our authorities. "Deep-seated or suppurative inflammation of the organ," is the form of its disease of which the diagnosis is most difficult. Mr. Martin presents us with the following description of the symptoms of this form :

"The disease is sometimes preceded by a perceptible falling off in the general health, such as emaciation, dry cough, and embarrassed respiration, loss of appetite, with a muddy or sallow complexion; but it more generally comes on in the midst of apparent health. We seldom, indeed, see the patient till inflammation has actually commenced; when there is generally present a feeling of abdominal uneasiness, but more particularly of the epigastric region, and that of the liver, with some degree of fever, preceded by slight rigor or ague; but all these are so trifling as too often to attract little of the patient's attention. Perhaps he applies to his physician on account of diarrhœa, supposed to be the result of error in diet; medicine affords some relief, and he proceeds in his ordinary occupations for days, or, when the action is more chronic for weeks, though under great depression of the mental and corporeal energies; till at length his altered appearance — hacking cough — permanently dry skin — invincibly rough furred tongue, and morbid taste; all expressive of a depressed and depraved state of the secretions and excretions, attract some more serious notice on his own part, or that of his family. The real nature of the disease may still remain a secret to both patient and physician, and it may not be till actual tumour of the liver, a marked succession of rigors, or profuse and clammy sweats announce in *audible* terms the formation of abscess, that either party becomes awake to the impending danger, and then it is too late. A sense of uneasiness amounting to dull pain, weight and oppression may or may not exist in the region of the liver, according as the disease is seated more or less deep in its substance or in its upper convex surface; when the former exists, the symptoms are more than usually obscure and insidious; in the latter they are acute." (Johnson and Martin, pp. 277-8.)

With regard to another symptom supposed by other authorities to be of some importance, Mr. Martin remarks, that Mr. Twining *fancied* that " a greater degree of tension of the right rectus abdominis muscle than of the left," formed " one of the most undeviating symptoms of congestion with incipient interstitial deposit into the texture of the liver, which generally goes on to deep-seated abscess." Mr. Martin argues, " that the mere tension of a muscle can prove an ' undeviating symptom' of any peculiar morbid change in a gland beneath it, cannot be admitted in any case." We would remark on this, that the late very able and excellent Mr. Twining did not, in the present instance, express the opinion that the tension of the muscle was an absolutely undeviating symptom of the affection, but that it was one of the symptoms which were the most so. In truth, in suppurative inflammation of the parenchyma of the liver, in which we have little or no pain, and there are not one or two decisive phenomena to guide us in our diagnosis, we attain to our conclusion from an assemblage of circumstances, each in itself of little weight, but constituting in their union, if not conclusive, strong presumptive evidence, and of this assemblage, the tension of the right *rectus abdominis*, we have found, even from observation in this country, as important as any. We find Dr. Maclean, too, in reporting a case of hepatic abscess explored and punctured, in the 2d volume of Madras Reports, page 232, quoting this symptom, " which Mr. Twining gives as a characteristic symptom of central abscess of the liver," as one of his reasons for the proceedings he adopted, exploring and puncturing the liver. The result was, the discharge of eight or nine ounces of thick, curdy pus.

We find that the exploratory needle is now extensively used in India, in supposed cases of hepatic abscess, having been first applied to this purpose by Dr. Mouat. The very able inspector of hospitals at Madras, Dr. Murray, seems to be exerting himself, and very successfully, in leading the medical staff there to decisive proceedings, in cases where there are good grounds for believing that abscess of the liver exists. From circumstances connected with the case already adverted to, that treated by Dr. Maclean, he is led to the conclusions, that danger need not be apprehended from pushing a trocar into the parenchymatous substance of the liver, and that such punctures are the means of causing adhesion between the liver and abdominal parietes. In his labour to surmount the reluctance of medical practitioners to explore enlarged livers, he remarks that a " *deterring*" story is told here of a patient once dying from hemorrhage in consequence of a trocar having been pushed into his liver; but he (Dr. Maclean) can call to mind seventeen cases within the last four years, wherein he performed this operation without any bad consequences; by which means, six of the number recovered. In continuation, he remarks, that with a good anatomical knowledge of the region in our mind's eye, to enable us to avoid the large hepatic vessels, the gall-bladder, the colon, and the stomach, there is abundance of evidence to authorise us, nay to render it our bounden duty, to explore the liver, without hesitation or delay, in most cases where pathognomonic symptoms of abscess in it exist, and the disease is interfering seriously and prejudicially with the functions of the organ, and the general health of the patient. Dr. Murray gives the following rules, on which we should place confidence, being convinced that they are the result of experience, for exploring and puncturing the liver. All our punctures, he thinks, should be made from the abdominal cavity, entering the trocar or explorer under the edge of the cartilages of the seventh, eighth, or ninth ribs, as circumstances may indicate. We may often get nearer the abscess through one of the intercostal spaces; and primary exploration may sometimes be advantageously made in this situation, by a very flat canular instrument; but from patients not having recovered when the matter was evacuated through the diaphragm; from the fibres of the diaphragm impeding the exit of the matter like a valve; from observing that air sometimes enters the wound when made here, and from the opening not being so dependent when made through the intercostal spaces as through the abdominal parietes, he is induced to prefer the latter mode in all cases. He prefers a long flat trocar for the performance of the operation.*

In corroboration of the value of a long trocar, surgeon Wilkins, of H. M. 41st regiment, relates a case of great interest, which will be found at page 479 of the first volume of the Madras Journal. In this case, two punctures had been made with a trocar in the intercostal spaces to no great depth. There was some effusion of blood, with a degree of relief, but no permanent benefit; no pus was discharged. Mr. Wilkins feeling a conviction that the common trocar was too short, on the 5th of April introduced that employed for puncturing the bladder by the rectum, which being curved and larger than the other, reached the abscess, and gave vent to four ounces of sanious purulent matter, to the great relief of the patient. On the 11th of the same month, the long trocar was again introduced, as the discharge had stopped; the punctures were kept open by tents; the discharge soon became healthy pus, which gradually lessened in quantity, till it finally ceased on the 10th of May, and the wounds soon healed. The patient was restored to perfect health.

Mr. Martin has employed very commendable pains in illustrating the relative frequency of liver disease in different districts. Prior to his investigations, this class of complaints was supposed to be very infrequent in Bengal; but his researches prove the fallacy of this supposition. He has received in his researches the very able assistance of Major Tulloch, to whom he acknowledges his obligations for a very valuable table on the subject, published in page 282 of his, and Dr. Johnson's work. From this valuable document, we find that, in the Windward and Leeward Command in the West Indies, of the average strength $44\frac{1}{2}$ were attacked with inflammation of the liver and jaundice, of whom died 1 in 12; and that in Jamaica of the garrison $95\frac{3}{5}$, of which was fatal 1 in 11, were attacked. When we turn to the eastern world, we observe, that in Madras the proportion attacked was 1 in 9 nearly, of which was fatal 1 in $17\frac{3}{4}$; in Bengal the proportion attacked was 1 in $15\frac{5}{6}$, of whom died 1 in 14; whilst in Bombay, the numbers attacked were 1 in $16\frac{1}{2}$, in which the fatality was 1 in $17\frac{1}{2}$.

From this table it appears that the fatality from a given number of cases

* Madras Journal, vol. ii. pp. 237-9. We learn with much regret, since this article was written, that Dr. Murray has since died in India.—ED.

of liver disease is greater in the western than the eastern hemisphere ; but that the proportion attacked with the disease is very much greater indeed in the latter than in the former locality. We know nothing of the cause of this great discrepancy, nor of that existing between the Windward and Leeward Command and Jamaica, where, it will be observed, not half the number are attacked that suffer in the former station. It must be manifest that difference of temperature will not explain it; neither will it explain the following singular anomalies pointed out by Mr. Martin: The deaths by hepatic diseases among the European troops in garrison at Fort William are stated in the returns furnished on the spot at 1 in 18, while at Chinsurah, but eighteen miles distant, they are 1 in 7 ; at Berhampore 1 in 9 ; at Winapore 1 in 6 ; and lastly, in the General Hospital, Calcutta, 1 in 9. Mr. Martin suspects there may be some error in the estimate for Fort William, and the difference between this and the other stations mentioned is so great, there seems some ground for the suspicion.

Chronic enlargement of the liver meets an ample and well-merited share of attention from our authors. The opinion expressed long ago by Dr. Johnson, that not 1 in 10 of those who are supposed to labour under chronic liver disease have any organic affection of that viscus, received, it is well-known, an ample confirmation from the dissections performed at Hilsea Barracks by Dr. Knox, from which it resulted that of from 40 to 60 bodies of persons supposed to be labouring under hepatities or hepatic dysentery, in 2 only were there any traces of organic disease of the liver. The opinion expressed by Dr. Johnson would naturally point to him as a very suitable instructor in disorders which simulate actual disease in the liver, and a very able chapter has he written on this subject, for which we refer our readers to the original. Actual chronic disease does, however, frequently exist, either as the result of previous inflammatory states, or congestion of the organ, or as the sequel of intermittent or remittent fever. The following is Mr. Martin's description of the symptoms :

"The liver is generally enlarged in a perceptible manner; its function is greatly impaired, the biliary secretion being scanty and depraved; that of the kidneys is similarly affected ; there is frequently a hacking dry cough, dyspepsia is various, and general ill health; sometimes a cachectic state with a sallow pasty complexion, and emaciation. The mind is despondent." (Johnson and Martin, p. 283.)

This is often an unmanageable disease. Mr. Martin believes mercury injurious, for it injures the stomach and bowels, without exciting secretion from the liver; and drastic purgatives are equally pernicious. His favorite remedy is the nitro-muriatic bath steadily persevered in for a month or six weeks at a time. Mr. Annesley entertains the same opinion of the value of this remedy after due depletion. He recommends that whether chronic inflammation continues as a consequence of the active form of hepatitis, or takes place primarily, local depletion should be practised, according to the state of the pulse and appearance of the tongue, and the habit and constitution of the patient. Simultaneously with such vascular depletion, the state of the biliary and intestinal discharges should be corrected by suitable purgatives, such, in the first instance, as calomel at bedtime followed by some saline aperients in the morning, and subsequently by the milder mercurials, blue pill or mercury with chalk, and a morning aperient. After the employment of these remedies, he would, equally with Mr. Martin, resort to the nitro-muriatic bath.

The following is the receipt for the preparation of the bath, and the mode of its employment recommended by Mr. Martin:

"Two gallons of water (about ten bottles) suffice for a bath. To each gallon of water, add 3 ounces of the dilute nitro-muriatic acid by measure. The bath thus prepared will keep in use for three days, by adding half an ounce of dilute acid and a pint of water, morning and evening, in order to make up for the waste by evaporation, a portion only of the bath is to be heated for use, after which it is to be added to the remainder, so as to make the whole of a comfortable warmth. Let both feet be placed in the bath, while the inside of the legs and thighs, the right side (over the liver,) and the inside of both arms are sponged alternately: this should be continued for ten or fifteen minutes morning and evening. Whilst using the bath, a gentle aperient such as Cheltenham salts, or Epsom salts in some bitter infusion, should be taken every other morning. Earthen or wooden vessels should be preferred as foot-baths, and all the sponges and towels to be kept in cold water, as the acid corrodes them." (Johnson and Martin, p. 286.)

A case is given illustrative of the beneficial effect of this remedy in the words of the patient, not in those of Mr. Martin, under whose care he was treated. It is so valuable that we give it a place here:

"In November, 1829, I arrived in Calcutta, suffering from the consequences of a jungle fever contracted at Chittagong; my liver and spleen were perceptibly enlarged, my limbs were much swollen and so stiff that I could with difficulty walk, and the least exercise occasioned vomiting. Before my arrival at the Presidency, I had for months taken medicine; this plan was altered, and I was put through a course of the nitric-acid bath, taking a vapour-bath every other day. The nitric-acid bath acted in a few days very powerfully immediately on using it; and in about three weeks both the liver and spleen could no longer be felt, nor did pressure give me much uneasiness; the stiffness too disappeared, and my skin became less tense and dry. I took an aperient draught once or twice a week, and nothing else but the bath. I left Calcutta towards the end of December, and had little or no occasion for medicine for two years afterwards, my general health being completely restored." (Ibid. p. 284.)

DISEASE OF THE SPLEEN. This affection forms an important object of attention to the tropical physician, and we do not regard it as so rare in this country as to merit neglect from him who stays at home. The subject is carefully and judiciously treated in both the systematic works before us. As a general rule, it arises from the same cause as intermittent and remittent fever, and more frequently in conjunction with one or the other of these fevers, or as a sequel of them than as a primary disease, though we have witnessed cases in this country unpreceded by fever of any kind, and under circumstances which appeared to exclude the idea of a malarious origin. It may exist either in an acute form with manifest indications of inflammation, or in that of chronic enlargement. How far the latter condition may arise independently of the former may be questioned; but, for our own parts, we would say that we have observed, even in this country, a state of chronic enlargement of the organ unpreceded by an inflammatory condition, and conversely we have seen this latter state subdued by appropriate treatment without the former ensuing.

The following is the result of Mr. Annesley's researches into the pathological condition of the organ.

"1. Enlargements of this viscus. The enlargements sometimes are to a very great extent, the spleen weighing ten or twelve pounds, and yet no sensible alteration can be detected in its substance by the unassisted eye. Frequently, however, its structure is at the same time much changed, its colour being much deeper, its consistence greatly diminished and rendered more friable, so as scarcely to admit of examination without falling to pieces. Its external membrane is also often torn with ease: sometimes it is thickened, more vascular, and occasionally cartilaginous in parts. 2. It is in some cases ossified in various places, and in others covered with large patches of coagulable lymph and albuminous concretions. In such cases, it frequently adheres to the adjoining surfaces and viscera. 3. Its internal structure frequently contains purulent collections, sometimes apparently unencysted, and flowing through its substance; at others, inclosed in one or more distinct cysts. It is also subject to tubercular formations, and to hydatids. 4. The spleen is sometimes found smaller than natural, and dry and shrivelled; but this is comparatively a rare occurrence in warm climates. 5. Instances have occurred wherein it has been ruptured from the congestion of blood to which it has been subject in the cold stage of an ague. And, 6th, its internal substance has been found reduced to a grumous and pultaceous mass." (Annesley, pp. 312-13.)

It will be understood that the treatment of the acute affection will be governed by different principles from that of chronic. In the former we are advised to employ bleeding, general or local, purgatives and sudorifics. To these remedies, adopted by Mr. Martin, Mr. Annesley would add mercurial purgatives, a mode of proceeding which has incurred some censure, it being observed that the peculiar cachectic condition of the system with which the chronic form of the affection is associated, supports ill this remedy. It is perfectly true that nothing could be considered more pernicious than the effect of mercury on the pallid and sanious state associated with non-inflammatory spleen disease; but in the acute inflammatory form we have ourselves employed mercury combined with sudorifics, conjointly with local bleeding, not only without prejudice, but with the effect of curing the patient.

The appearance of one labouring under chronic disease of the spleen is concisely and graphically drawn by Mr. Martin, when he says, "the complexion is of a dirty lemon colour, the countenance being puffed and bloated; the eye full, and of a peculiar clearness; the lips and tongue blanched and bloodless; in short we have a concentration of the cachexia of systematic writers." (p. 297.) This state, resembling the anemious condition induced by hemorrhage, and existing in chlorosis, on which we observed on a former occasion the spleen was very generally affected,* is what we ever find associated with chronic affections of the same organ in this country,—a state so naturally suggesting the tonic and astringent plan of treatment, that such a one has been adopted from the days of Celsus, when "*incoctum absinthium*" was the remedy administered in the morning, and "*aqua à ferrario fabro, in quâ condens ferrum subinde tinctum sit*," was the post-prandial potion, to the present time, when Shoolbred's powder, a combination of purgatives with sulphate of iron is the universal nostrum in Calcutta for the disease. Mr. Annesley, besides the employment of purgatives with tonics, suggests the internal administration of nitric acid, and the application of the nitro-muriatic lotion over the region of the spleen. Mr. Martin finding that, when muco-intestinal irritation is present with a hectic form of fever, the spleen medicines in common use inadmissible, recommends a combination of tincture of muriate of iron and tincture of iodine in equal parts, of which ten drops are

* See British and Foreign Med. Rev., vol. III. p. 346.

given three times a day, the spleen being at the same time rubbed with croton oil. When the tincture is found to disagree, he recommends pills of the iodide of lead according to the following form :

℞ Plumbi iodidi ʒss.
Confectionis rosæ q.s.s. Divide in pilulas cxliv.

Of these pills one is given night and morning, increasing the number gradually.

We have brought our review of these valuable works to a close. Our estimate of their worth has been so clearly indicated in the course of this article, that to enlarge on their very great merits here would be superfluous. To the two systematic works, at least, the seal of public approbation being already affixed, they can require no commendation from us. We will only say that Dr. Johnson's volume, always excellent, has been much improved by himself in the present edition; while the numerous important additions made to it by Mr. Martin have greatly enhanced its value as a guide to the tropical practitioner. We must take this opportunity also of bearing testimony to the merits of the different Indian journals whose titles we have prefixed to this article. Of the Madras Journal, more especially, from which we have derived much information, we must speak in high terms. The spirit, intelligence, and method displayed in it are equally creditable to the editor and the contributors; and we wish it the ample success to which these high qualities entitle it.

---

